# Harnessing the immunotherapeutic potentials of gamma delta T cells against hematological malignancies

**DOI:** 10.1002/hem3.70182

**Published:** 2025-08-07

**Authors:** Charles Agbuduwe, John Maher, John Anderson

**Affiliations:** ^1^ Centre for Haematology Imperial College London London UK; ^2^ Catherine Lewis Centre (Haematology), Hammersmith Hospital Imperial College Healthcare NHS Trust London UK; ^3^ School of Cancer and Pharmaceutical Sciences Kings College London London UK; ^4^ Department of Immunology Eastbourne Hospital Eastbourne East Sussex UK; ^5^ Zayed Centre for Research and The UCL Great Ormond Street Institute of Child Health University College London London UK

## Abstract

Gamma delta (γδ) T cells, which constitute about 5%–10% of peripheral blood lymphocytes, play key roles in tumor immunosurveillance and are often enriched within epithelial tissues. They are unique in their Major Histocompatibility Complex‐independent antigen recognition via the γδ T‐cell receptor (TCR) as well as via innate receptors, making them ideal1 candidates for allogeneic “off‐the‐shelf” cell therapy products. In humans, two main structural subsets of γδ T cells—Vδ1 and Vδ2—have been defined, which differ in TCRδ chains, effector function, and tissue localizations. Vδ2 T cells constitute the majority of γδ T cells in peripheral blood and can be expanded with aminobisphosphonates such as zoledronic acid. In recent years, the potent antitumor functions of Vδ1 T cells have also been recognized, and new expansion protocols are being developed. Given the ample preclinical evidence of γδ T‐cell efficacy against hematological malignancies, several γδ T‐cell‐based cell therapy products are currently in clinical development, and there has been an exponential increase in the number of adoptive γδ T‐cell therapy clinical trials. This comprehensive review provides an overview of the rationale for γδ T‐cell therapy, ongoing clinical trials, as well as the challenges and future role of γδ T‐cell‐based immune therapies in hematology.

## BACKGROUND

Gamma delta (γδ) T cells usually constitute approximately 5%–10% of peripheral blood (PB) T cells and are a distinct lineage of T lymphocytes which express T‐cell receptors (TCRs) composed of gamma (γ) and delta (δ) chains.^1^ The majority of PB T lymphocytes express the αβTCR which usually recognizes peptides presented in the context of self‐Major Histocompatibility Complex (MHC) molecules with co‐receptors CD4 or CD8 required for αβ T‐cell activation. Unlike αβ T cells, antigen recognition by γδ T cells is usually not MHC‐restricted and γδ TCRs can recognize a wide range of antigens including small molecules, lipids, and whole proteins without the requirement for co‐receptor interaction.^2^, ^3^, ^4^ Some classical MHC‐restricted γδ TCRs have been identified^5^ but these are a small minority. In addition to antigen recognition via the TCR, γδ T cells possess innate receptors which recognize antigens associated with cellular stress. Therefore, γδ T cells belong to a unique group of unconventional T cells with innate and adaptive immune functions, capable of clonal expansion and rapid activation in response to antigen stimulation without prior sensitization.^6^ Although identified 40 years ago,^7^, ^8^ the crucial functions of these immune cells in health and disease are only being unraveled.

Based on the structure of the variable domains of the δ chains, two major subsets of γδT cells have been defined in humans (summarized in Table 1). Vδ2 chains almost invariably associate with Vγ9 chains and are thus referred to as Vγ9Vδ2 T cells. They are the best characterized γδ T‐cell subtype and are most predominant in PB. By contrast, Vδ1 T cells are more frequent in the skin and within epithelial tissues. Other subsets including Vδ3 T cells, which are enriched in the liver^9^ and gut epithelium,^10^ are rare. Relatively little is known about Vδ3 T‐cell function but recognition of the Major Histocompatibility Complex Class I‐related molecule 1 (MR1) has been reported for the Vδ3 TCR.^11^ The tissue localization of specific subsets suggests a vital role for γδ T cells in host defense and immune surveillance. Indeed, mice lacking γδ T cells demonstrated impaired clearance of bacterial infections^12^, ^13^, ^14^ due to delayed recruitment of immune cells, impaired mucosal immunoglobulin responses,^15^ and rapid development of skin tumors following exposure to carcinogens.^16^

**Table 1:** Characteristics of the major gamma delta (γδ) T‐cell subsets in humans.

γδ T‐cell subset	Tissue distribution	Phenotypic characteristics	Expansion methods
Vδ1	Enriched in skin, gut, and other epithelial tissues. Minor γδ population in peripheral blood.	Memory‐like features; clonotypic TCR focusing and rapid clonal expansion in response to antigenic stimulation. Recognize a wide range of ligands which are mostly undefined. Known ligands include MHC‐like molecules including MICA/B, CD1d. Cytolysis of cancer cell lines. Expression of NKRs. Relatively resistant to activation‐induced cell death. Higher expression of PD‐1 and TIGIT.	Anti‐CD3, Vδ1 agonist antibodies, and stimulatory cytokines.
Vδ2 (also Vγ9Vδ2)	Predominant in peripheral blood.	Semi‐invariant TCR pairing with Vγ9 chains. Recognition of intracellular accumulation of phosphoantigens in “stressed” cells. Professional antigen‐presentation function. Cytolysis of cancer cell lines. Expression of NKRs.	Zoledronate and stimulatory cytokines including IL‐2 and/or IL‐15.
Vδ3	Liver and gut epithelium. Rare in peripheral blood.	Little known; some MR1 reactivity and IL‐17 production.	Not defined.

Abbreviations: IL, interleukin; MHC, Major Histocompatibility Complex; MICA/B, MHC Class I chain‐related protein A and B; MR1, Major Histocompatibility Complex Class I‐related molecule 1; NKR, natural killer‐cell receptor; PD‐1, programmed cell death 1 receptor; TCR, T‐cell receptor; TIGIT, T‐cell immunoreceptor with immunoglobulin and ITIM domain.

Although the structure of the γδTCR suggests the potential of binding to a broad range of antigens,^17^, ^18^ the majority of γδTCR ligands remain elusive. The best characterized ligand of the Vγ9Vδ2 TCR are non‐peptide phosphoantigens (PAgs), intermediates of the isoprenoid (mevalonate) biosynthetic pathway.^19^ The most potent known pAg activator of the Vγ9Vδ2 TCR is the microbial precursor of isopentenyl pyrophosphate (IPP), (*E*)‐4‐hydroxy‐3‐methyl‐but‐2‐enyl pyrophosphate (HMB‐PP), produced by gram‐negative bacteria and *Mycobacterium tuberculosis*.^20^ In human cells, accumulation of IPP generated via both the classical and alternative mevalonate pathways occurs in states of cellular stress and in transformed cells.^21^ The inhibition by aminobisphosphonates of farnesyl pyrophosphate synthase, a key enzyme in the mevalonate pathway which converts IPP to farnesyl pyrophosphate, leads to intracellular accumulation of IPP and is the basis of many expansion methods for Vγ9Vδ2 T cells.^22^ The mechanism of Vγ9Vδ2 TCR recognition of intracellular IPP accumulation has been the subject of intense study in recent years. Crucial to this mechanism are the butyrophilins (BTNs), a family of Type I membrane proteins with two immunoglobin‐like extracellular domains and structural homology to the B7 superfamily of proteins.^23^ Intracellular PAgs bind to the intracellular B30.2 domain of BTN3A1 inducing a conformational change^24^, ^25^, ^26^ which permits it to associate with BTN2A1.^27^ The resulting BTN3A1–BTN2A1 complex then binds to and activates the Vγ9Vδ2 TCR.^27^, ^28^ While BTN2A1 binds directly to germline‐encoded regions of the Vγ9 chain,^29^ both BTN3A1 and BTN2A1 are required for TCR activation and the loss of BTN2A1 cannot be compensated for by BTN3A1.^28^ Recognition of the role of BTNs in the activation of Vγ9Vδ2 T cells has led to the development of an activating humanized BTN3A antibody which is currently in early phase clinical trials against a range of cancer types.^30^ In contrast to Vδ2 T cells, most ligands of the Vδ1 TCR are unknown but several putative candidates have been identified. These include several MHC superfamily members such as CD1d, which presents lipid molecules^31^, ^32^ and MHC Class I chain‐related protein A and B (MICA/B).^6^ MICA/B are also ligated by natural killer Group 2D (NKG2D) which is expressed by both Vδ1 and Vδ2 T cells. The expression of such innate receptors in addition to TCR specificity make γδ T cells robust immune effectors.

Besides the difference in antigen recognition and tissue localization between Vδ1 and Vδ2 γδ T cells, several phenotypic distinctions have been observed. In contrast to Vδ2, the Vδ1 subset expresses higher levels of C–C chemokine receptor Type 7 (CCR7) and chemokine (CXC) receptor (CXCR)−1, whereas there is higher C–C chemokine receptor Type 5 (CCR5) expression in the former.^33^, ^34^ These differences in the expression of homing receptors have been suggested as a possible mechanism for the differential tissue localizations of γδ T‐cell subsets. Vδ1 T cells also express higher levels of PD‐1 compared to Vδ2 T cells^35^; however, recently published work has shown that PD‐1‐expressing Vδ1 T cells represent a functional, tissue‐adapted state, which is in contrast to the association of PD‐1 upregulation with functional exhaustion in cytotoxic αβ T cells.^36^ Vδ1 T cells are more resistant than Vδ2 T cells to the immunosuppressive effects of galectin‐3^37^ and activation‐induced cell death.^34^ Recognition of the putative tissue adaptations and immunotherapeutic potentials of Vδ1 T cells has triggered recent interests in specific expansion methods for this subset.

## ANTICANCER FUNCTIONS OF γδ T CELLS

**Figure 1:**
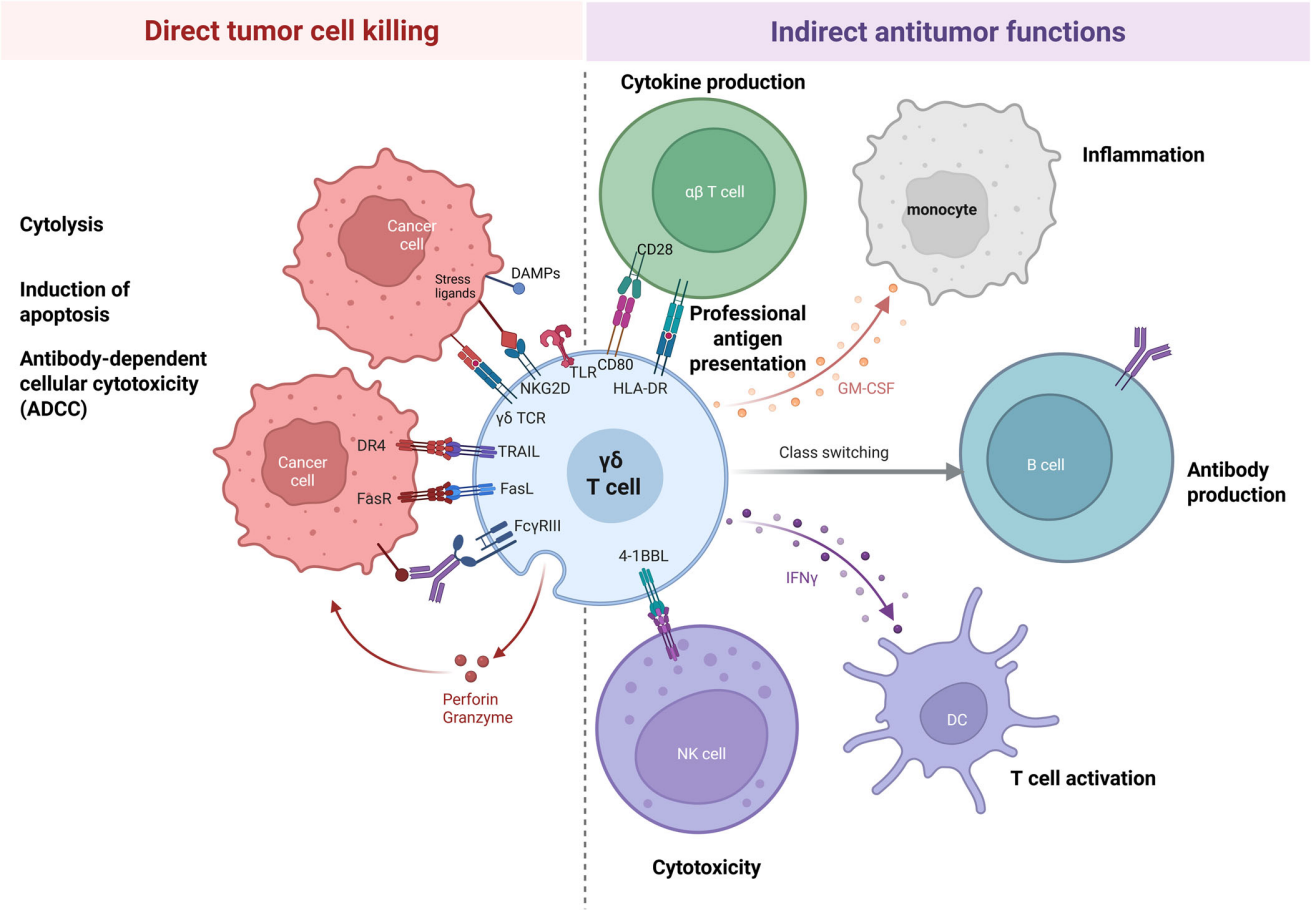
**Schematic illustration of the anticancer properties of gamma delta (γδ) T cells.** γδ T cells sense a wide range of cellular signals via innate receptors such as the natural killer Group 2D (NKG2D) receptor, which is activated by a range of stress signals and Toll‐like receptors, which sense DAMPs, damage‐associated molecular patterns. Target cell engagement via the γδ TCR is also achieved via a range of ligands that are upregulated in malignant cells and other states of cellular stress. The antitumor activity of γδ T cells occurs via both direct mechanisms including the release of cytolytic enzymes such as perforin and granzyme, induction of apoptosis via the Fas ligand (FasL) or tumor necrosis factor‐related apoptosis‐inducing ligand (TRAIL) as well as via antibody‐dependent cellular cytotoxicity. Indirect antitumor mechanisms include activation of other immune cell mediators such as macrophages and dendritic cells (DCs) via secretion of chemokines such as interferon‐γ (IFNγ), granulocyte‐macrophage colony‐stimulating factor (GM‐CSF). γδ T cells also act as professional antigen‐presenting cells to αβ T cells via the Class II Major Histocompatibility Complex molecule, HLA‐DR, and by providing co‐stimulation via the CD80‐CD28 axis. Class switching of B cells to stimulate antibody production is induced by γδ T cells via secretion of IL‐4 and IL‐10. Activation of NK cells also occurs via the 4‐1BB:4‐1BB ligand (4‐1BBL) interaction. DR4, death receptor 4; FasR, Fas receptor; FcγRIII, Fc‐gamma receptor III. This figure was created with Biorender.com.

γδ T cells mediate anticancer responses via direct and indirect mechanisms (summarized in Figure 1). Upon activation via the γδ TCR and/or other activating receptors including NKG2D, CD94/NKG2C, DNAX accessory molecule‐1 (DNAM‐1), NKp30, and NKp44,^38^ γδ T‐cells can initiate lysis of target cells by secretion of cytolytic proteins such as perforins and granzyme B.^39^, ^40^ Experimental evidence from receptor blocking suggests that γδ T cells can lyse target cells following ligation of NKG2D, independent of TCR stimulation but lysis of some cancer cell lines requires signaling via the TCR in addition to NKG2D.^41^ An alternative pathway for direct cell lysis involves the initiation of apoptosis of target cells via the interaction with FasL and tumor necrosis factor (TNF)‐related apoptosis‐inducing ligand (TRAIL) with their respective receptors.^42^ Furthermore, γδ T cells express the low‐affinity Fc‐gamma receptor CD16 (FcγRIII), which can initiate antibody‐dependent cellular cytotoxicity (ADCC). Experimental evidence of ADCC is supported by the observation that γδ T‐cell cytotoxicity is enhanced following the addition of rituximab and trastuzumab to cultures with CD20+ and HER2+ target cells, respectively.^43^ γδ T cells also mediate anticancer responses indirectly by the activation and recruitment of other immune cells via secretion of cytokines and acting as professional antigen presenting cells (APCs).^44^ Given their versatility and lack of MHC restriction, γδ T cells are excellent candidates for adoptive cell therapy against cancers particularly as an off‐the‐shelf product.

## EXPERIMENTAL EVIDENCE OF γδ T‐CELL ACTIVITY IN HEMATOLOGICAL MALIGNANCIES

The antitumor reactivity of γδ T cells has been demonstrated in several experimental models of hematological malignancies. Early in vitro experiments demonstrated effector functions for unfractionated γδ T cells activated with IL‐2, against cancer cell lines including the Daudi Burkitt's lymphoma‐derived cell line, which lacks expression of MHC Class I.^45^ The observation that the addition of blocking antibodies against HLA A, B, and C did not diminish their cytotoxic activity first gave credence to the MHC‐independent mode of antigen recognition by γδ T cells.^46^ While most early in vitro experiments demonstrating anticancer properties of γδ T cells involved unfractionated (predominantly Vδ2 subset), the Vδ1 subset has also been shown to possess potent anticancer properties. γδ T cells of the Vδ1 subset expanded with concanavalin A (Con A) demonstrated specific cytotoxicity against chronic lymphocytic leukemia (CLL) cells in vitro.^47^ Similarly, phytohemagglutinin (PHA)‐expanded Vδ1 T cells were cytotoxic against myeloma cell lines and primary bone‐marrow‐derived malignant plasma cells.^48^ More recently, clinically relevant methods based on cytokines and agonist antibodies have been developed for the expansion of the Vδ1 subset. Purified Vδ1 T cells expanded with a cocktail of cytokines (“DOT cells”) were cytotoxic against CLL cell lines, autologous and allogeneic primary CLL cells but spared autologous healthy leukocytes.^49^ A simple expansion method using anti‐CD3 (OKT3) and IL‐15 resulted in the expansion of Vδ1 T cells, which were cytotoxic against neuroblastoma and leukemia cell lines in vitro but not reactive against allogenic PBMCs.^50^

One of the earliest clinical indications of the role of γδ T cells in immune surveillance was from the observation of an association between improved leukemia free survival (LFS) and increased γδ T‐cell frequency (>10% of T cells in PB) following partial T cell depleted allogeneic stem cell transplantation (SCT) for acute leukemia.^51^ An update of the study, including long‐term (8‐year) follow‐up, confirmed that T‐cell recovery with increased γδ T cells (as opposed to normal or low levels) was associated with significantly improved 5‐year overall survival (OS) (70.8% vs. 19.6%, P < 0.0001) and 5‐year LFS (54.4% vs. 19.1%, P = 0.0003). No increased incidence of acute graft versus host disease (GVHD) was observed in the high γδ T‐cell frequency group. Similar findings were observed in the pediatric post‐SCT setting (*n* = 102).^52^ Notably, in addition to improved event‐free survival, elevated γδ T‐cell reconstitution was associated with a significantly decreased cumulative incidence of infections (0.18% vs. 0.53%; P = 0.02). An analysis from cohorts of newly diagnosed acute myeloid leukemia (AML) patients identified an association between low Vγ9Vδ2 T‐cell frequency and significantly inferior 5‐year overall and relapse‐free survivals.^53^ Furthermore, Gentles et al. analyzed genomic signatures from about 18,000 tumors and 39 malignancies, identifying tumor‐infiltrating γδ T cells as a strong positive predictor of survival.^54^ These findings provide strong evidence for the role of γδ T cells in tumor immunosurveillance. Furthermore, their MHC‐independent mechanism of antigen recognition permits graft versus leukemia (GvL) effects without the undesirable GVHD response. Based on the lack of alloreactivity of γδ T cells and prior observations of the reduced risk of disease recurrence post‐allogeneic SCT, the adoptive transfer of ex vivo expanded donor‐derived γδ T cells has become an attractive strategy that is being investigated in clinical trials.

## CLINICAL TRIALS OF γδ T‐CELL‐BASED CELL THERAPY IN HEMATOLOGICAL MALIGNANCIES

**Figure 2:**
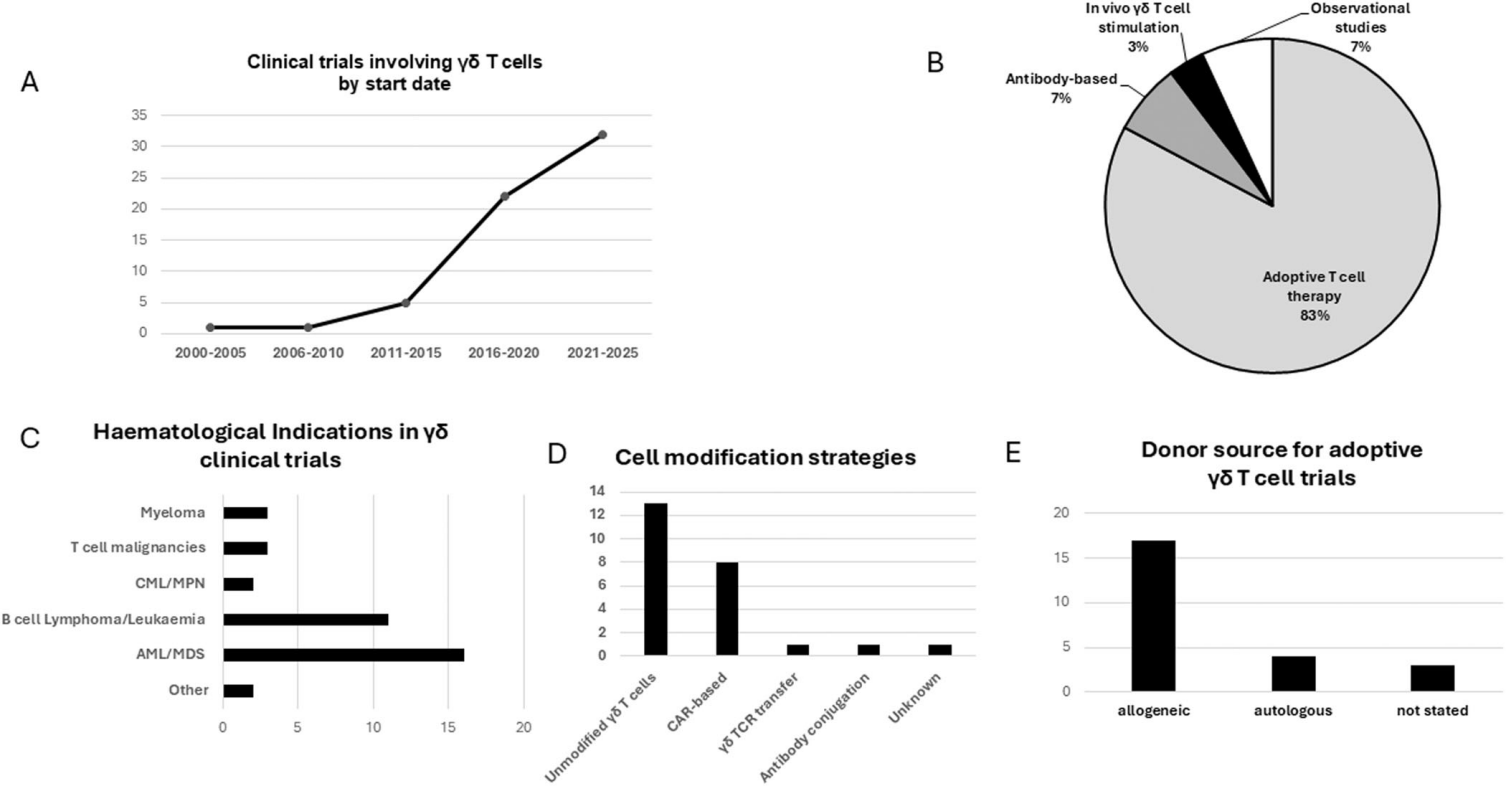
**Summary of gamma delta (γδ) T cells based clinical trials. (A)** Number of γδ T‐cell‐related clinical trials by start date. **(B)** Proportions of study types for hematology‐related γδ T‐cell trials. **(C)** Hematological indications in γδ T‐cell‐related clinical trials. **(D)** Cell modification strategies of γδ T‐cell‐related clinical trials in hematology. **(E)** Donor source for adoptive γδ T‐cell clinical trials in hematology. Clinical trial details were accessed via clinicaltrials.gov as of January 31, 2025. AML, acute myeloid leukemia; CML, chronic myeloid leukemia; MDS, myelodysplastic syndrome; MPN, myeloproliferative neoplasm.

Early attempts at in vivo stimulation of Vγ9Vδ2 T cells with the administration of aminobisphosphonates and IL‐2 to patients achieved variable expansions, limited clinical responses, and toxicities arising from systemic cytokine effects.^55^, ^56^ More recently, there has been a surge in the number of registered clinical trials of adoptively infused γδ T cells (Figure 2). Although the majority of current γδ T‐cell therapy trials are for solid malignancies, this review will focus on clinical studies for hematological indications. There were 29 ongoing, recently completed, or terminated clinical trials involving γδ T cells for hematological conditions listed on clinicaltrials.gov (accessed January 31, 2025), and these are summarized in Table 2. The majority of γδ T‐cell‐related clinical studies were adoptive cell therapy strategies, and the most frequent hematological indication was AML, followed by B‐cell malignancies (Figure 2).

**Table 2:** Gamma delta T‐cell‐based clinical trials in hematology.

NCT number	Study title	Acronym	Other IDs	Study status	Conditions	Interventions	Cell type	Donor type	Cell modification?	Sponsor	Phases	Funder type	Study type	Start date
*Adoptive cell therapies*														
NCT05886491	A study of GDX012 in adults with relapsed or refractory acute myeloid leukemia		TAK‐012‐1501	RECRUITING	AML	BIOLOGICAL: GDX012	Vδ1‐enriched γδ cells	Allogeneic	No	Takeda	PHASE 1/2	INDUSTRY	INTERVENTIONAL	2023
NCT05686538	Innate donor effector allogeneic lymphocyte infusion after stem cell transplantation: the IDEAL trial	IDEAL	Protocol VIP14.0.11	RECRUITING	AML/MDS	OTHER: innate donor lymphocyte infusion (iDLI)	TCR αβ/CD19 depleted DLI with >10^6^ γδ T cells/kg	Allogeneic	No	Rigshospitalet, Denmark	PHASE 2/3	OTHER	INTERVENTIONAL	2022
NCT05653271	ACE1831 in adult subjects with relapsed/refractory CD20‐expressing B‐cell malignancies		ACE1831‐001	RECRUITING	B‐cell lymphoma; NHL, DLBCL, PMBCL, MZL, FL	BIOLOGICAL: ACE1831|DRUG: obinutuzumab	γδ T cells conjugated to anti‐CD20 antibody via DNA linkers	Allogeneic	Yes, CD20 antibody conjugation	Acepodia Biotech, Inc.	PHASE 1	INDUSTRY	INTERVENTIONAL	2023
NCT05554939	Allogenic CD19‐targeting CAR‐γδ T cell therapy in r/r NHL		CHN‐PLAGH‐BT‐072	RECRUITING	NHL	BIOLOGICAL: allogenic CD19‐CAR‐γδ T cell	γδ T cells	Allogeneic	Yes, CD19‐CAR	Chinese PLA General Hospital	PHASE 1/2	OTHER	INTERVENTIONAL	2022
NCT05388305	Universal CAR‐γδ T cell injection in the AML patients		CAR‐γδ T for AML	UNKNOWN	AML	BIOLOGICAL: CAR‐‐ γδT cells	γδ T cells	Unknown	Yes, CAR‐transduced	Hebei Senlang Biotechnology Inc., Ltd.	NA	INDUSTRY	INTERVENTIONAL	2022
NCT05358808	ACHIEVE ‐ efficacy and effectiveness of adoptive cellular therapy with ex‐vivo expanded allogeneic γδ T‐lymphocytes (TCB008) for patients with refractory or relapsed acute myeloid leukaemia (AML)	ACHIEVE	TCB008‐001	RECRUITING	AML, MDS	BIOLOGICAL: TCB008	Vγδ92 T cells	Allogeneic	No	TC Biopharm	PHASE 2	INDUSTRY	INTERVENTIONAL	2022
NCT06463327	ACHIEVE2 ‐ safety and preliminary efficacy of intravenous TCB008 in patients with relapse or refractory acute myeloid leukemia (AML) or myelodysplastic syndrome (MDS)/AML	ACHIEVE2	TCB008‐003	NOT YET RECRUITING	AML, MDS	BIOLOGICAL: TCB008	Vγ9δ2 T cells	Allogeneic	No	TC Biopharm	PHASE 1	INDUSTRY	INTERVENTIONAL	2025
NCT05302037	Allogeneic NKG2DL‐targeting CAR‐γδ T cells (CTM‐N2D) in advanced cancers (ANGELICA)	ANGELICA	CTM‐N2D‐102	RECRUITING	Refractory/relapsed cancer	BIOLOGICAL: allogeneic NKG2DL‐targeting chimeric antigen receptor‐grafted γδ T cells (CTM‐N2D)	γδ T cells	Allogeneic	Yes, NKG2DL‐CAR	CytoMed Therapeutics Pte Ltd.	PHASE 1	INDUSTRY	INTERVENTIONAL	2024
NCT05015426	Gamma delta T‐cell Infusion for AML at high risk of relapse after Allo HCT		MCC‐20305|9BC08	ACTIVE NOT RECRUITING	AML	BIOLOGICAL: gamma delta T‐cell infusion	γδ T cells	Allogeneic	No	H. Lee Moffitt Cancer Center and Research Institute	PHASE 1	OTHER	INTERVENTIONAL	2022
NCT05001451	Study of GDX012 in patients with MRD positive AML		GDX012U‐001	TERMINATED (BUSINESS DECISION)	AML	BIOLOGICAL: GDX012 suspension for IV Infusion	Vδ1‐enriched γδ cells	Allogeneic	No	GammaDelta Therapeutics Limited	PHASE 1	INDUSTRY	INTERVENTIONAL	2021
NCT04887259	Trial of LAVA‐051 in patients with relapsed/refractory CLL, MM, or AML		LV2020‐001	TERMINATED	CLL, MM, AML	BIOLOGICAL: LAVA‐051|BIOLOGICAL: interleukin 2	γδ T cells	Unknown	Yes, CAR‐transduced	Lava Therapeutics	PHASE 1	INDUSTRY	INTERVENTIONAL	2021
NCT04796441	Clinical study of universal CAR‐γδ T cell injection in the treatment of patients with relapsed AML after transplantation		CAR‐Î³Î´T cell for AML	UNKNOWN	AML	BIOLOGICAL: CAR‐γδ T	γδ T cells	Unknown	Yes, CAR‐transduced	Hebei Senlang Biotechnology Inc., Ltd.	NA	INDUSTRY	INTERVENTIONAL	2020
NCT04764513	Safety and efficiency of γδ T cell against hematological malignancies after allo‐HSCT		CHN‐PLAGH‐BT‐062	RECRUITING	AML, ALL, MDS, lymphoma	BIOLOGICAL: ex vivo expanded γδ T‐cell infusion	γδ T cells	Allogeneic	No	Chinese PLA General Hospital	PHASE 1/2	OTHER	INTERVENTIONAL	2021
NCT04735471	A phase 1 study of ADI‐001 in B cell malignancies	GLEAN‐1	ADI‐20200101	ACTIVE NOT RECRUITING	FL, MCL, MZL, PMBCL, DLBCL, NHL	BIOLOGICAL: ADI‐001	(Vδ1) γδ T cells	Allogeneic	Yes, anti‐CD20 CAR	Adicet Therapeutics	PHASE 1	INDUSTRY	INTERVENTIONAL	2021
NCT04696705	Allogeneic γδ T cells immunotherapy in r/r non‐Hodgkin's lymphoma (NHL) or peripheral T cell lymphomas (PTCL) patients		IIT2020028‐EC‐3	UNKNOWN	NHL, PTCL	BIOLOGICAL: ex vivo expanded allogeneic γδ T cells	γδ T cells	Allogeneic	No	Institute of Hematology & Blood Diseases Hospital, China	EARLY PHASE 1	OTHER	INTERVENTIONAL	2020
NCT04008381	Ex‐vivo expanded γδ T lymphocytes in patients with refractory/relapsed acute myeloid leukaemia		WHUH‐2009‐V010	UNKNOWN	AML	BIOLOGICAL: ex vivo expanded γδ T lymphocytes	γδ T cells	Allogeneic	No	Wuhan Union Hospital, China	PHASE 1	OTHER	INTERVENTIONAL	2019
NCT03939585	Pre‐emptive infusion of donor lymphocytes depleted of TCR + T cells + CD19+ B cells following ASCT		CASE1Z19	WITHDRAWN	Allogeneic stem cell transplant candidate; AML, ALL, MDS, MPN, LPD	BIOLOGICAL: cellular therapy product	γδ T‐ and NK‐cell‐enriched (TCR αβ‐depleted)	Allogeneic	No	Leland Metheny	PHASE 1	OTHER	INTERVENTIONAL	2024
NCT03790072	Ex‐vivo expanded γδ T‐lymphocytes (OmnImmuneÂ®) in patients with acute myeloid leukaemia (AML)		TCB‐202‐001|2018‐000409‐22	COMPLETED	AML	BIOLOGICAL: OmnImmuneÂ®	γδ T cells	Allogeneic	No	TC Biopharm	PHASE 1	INDUSTRY	INTERVENTIONAL	2018
NCT03533816	Expanded/activated gamma delta T‐cell infusion following hematopoietic stem cell transplantation and post‐transplant cyclophosphamide		IIT‐2018‐gamma‐delta T cell	RECRUITING	AML, CML, ALL, MDS	BIOLOGICAL: EAGD T‐cell infusion	γδ T cells	Allogeneic	No	University of Kansas Medical Center	PHASE 1	OTHER	INTERVENTIONAL	2020
NCT02656147	Immunotherapy with CD19 CAR γδ T‐cells for B‐cell lymphoma, ALL and CLL		Doing‐004	UNKNOWN	Leukemia, lymphoma	BIOLOGICAL: anti‐CD19‐CAR γδ T	γδ T cells	Allogeneic	Yes; CD19 CAR	Beijing Doing Biomedical Co., Ltd.	PHASE 1	INDUSTRY	INTERVENTIONAL	2017
NCT05237206	Study of SUPLEXA in patients with metastatic solid tumours and haematologic malignancies		SUPLEXA‐101	COMPLETED	Oncology	BIOLOGICAL: SUPLEXA	NK, NK‐T, and T cells	Autologous	Unknown	Alloplex Biotherapeutics Inc	PHASE 1	INDUSTRY	INTERVENTIONAL	2022
NCT04702841	CAR‐γδ T cells in the treatment of relapsed and refractory CD7 positive T cell‐derived malignant tumors		2019‐ky012	UNKNOWN	CD7‐positive T‐cell malignancies	DRUG: chimeric antigen receptor modified γδ T cells	γδ T cells	Autologous	Yes, CD7 CAR	PersonGen BioTherapeutics (Suzhou) Co., Ltd.	EARLY PHASE 1	INDUSTRY	INTERVENTIONAL	2020
NCT04688853	A study to investigate the safety and efficacy of TEG002 in relapsed/refractory multiple myeloma patients		TEG002_MM_US_01	UNKNOWN	MM	BIOLOGICAL: TEG002	T cells	Autologous	Yes, TCRγδ‐transduced	Gadeta B.V.	PHASE 1	INDUSTRY	INTERVENTIONAL	2021
NCT04028440	γδ T cells immunotherapy in patients with relapsed or refractory non‐Hodgkin's lymphoma (NHL)		QT2019001‐EC‐2	UNKNOWN	NHL, CLL, PTCL	BIOLOGICAL: autologous γδ ´T cells	γδ T cells	Autologous	No	Institute of Hematology & Blood Diseases Hospital, China	EARLY PHASE 1	OTHER	INTERVENTIONAL	2019
*Antibody‐based therapies*														
NCT04887259	Trial of LAVA‐051 in patients with relapsed/refractory CLL, MM, or AML		LV2020‐001	TERMINATED (BUSINESS DECISION)	CLL, MM, AML	BIOLOGICAL: LAVA‐051|BIOLOGICAL: interleukin 2	N/A, CD1d/Vδ2 TCR bispecific antibody	N/A	N/A	Lava Therapeutics	PHASE 1	INDUSTRY	INTERVENTIONAL	2021
NCT04243499	First‐in‐human study of ICT01 in patients with advanced cancer	EVICTION	ICT01‐101	RECRUITING	Solid tumor, adult hematopoietic/lymphoid cancer	BIOLOGICAL: IV ICT01 (BTN3A antibody)	N/A antibody‐based	N/A	N/A	ImCheck Therapeutics	PHASE 1/2	INDUSTRY	INTERVENTIONAL	2020
*In vivo stimulation of gamma delta T cells*														
NCT03862833	Zoledronic acid in combination with interleukin‐2 to expand Vγ9δ2 T cells after T‐replete haplo‐identical allotransplant	HILDEGAZ	RC18_0419	COMPLETED	Hematopoietic stem cell transplantation	DRUG: IL2|DRUG: zoledronic acid	N/A	N/A	N/A	Nantes University Hospital	PHASE 1	OTHER	INTERVENTIONAL	2019
*Observational studies*														
NCT03885076	Gamma delta T cells in AML		CCR4877	UNKNOWN	AML	PROCEDURE: blood collection and bone marrow aspirate	N/A	N/A	N/A	Royal Marsden NHS Foundation Trust	N/A	OTHER	OBSERVATIONAL	2018
NCT04911478	Long‐term follow‐up study of allogeneic gamma delta (γδ) CAR T cells		ADI‐20200102	ENROLLING BY INVITATION	FL, MCL, MZL, PMBCL, DLBCL, NHL	BIOLOGICAL: ADI‐001	γδ T cells	Allogeneic	Yes, anti‐CD20 CAR	Adicet Therapeutics	N/A	INDUSTRY	OBSERVATIONAL	2022

*Note*: The list was obtained from clinicaltrials.gov on January 31, 2025.

Abbreviations: γδ, gamma delta; ALL, acute lymphoblastic leukemia; AML, acute myeloid leukemia; CAR, chimeric antigen receptor; CLL, chronic lymphocytic leukemia; CML, chronic myeloid leukemia; DLBCL, diffuse large B‐cell lymphoma; FL, follicular lymphoma; LPD, lymphoproliferative disorder; MCL, mantle cell lymphoma; MDS, myelodysplastic syndrome; MM, multiple myeloma; MPN, myeloproliferative neoplasm; MZL, marginal zone lymphoma; NCT, national clinical trial number; N/A, not applicable; NHL, non‐Hodgkin lymphoma; NK, natural killer; PMBCL, primary mediastinal B‐cell lymphoma; PTCL, peripheral T‐cell lymphoma; TCR, T‐cell receptor.

### Clinical studies involving in vivo γδ T‐cell stimulation

Several ongoing clinical trials employ strategies for activating γδ T cells in vivo including the administration of zoledronic acid (ZOL), cytokines, and/or activating antibodies. The feasibility of in vivo generation and stimulation of γδ T cells was investigated in the Phase 1 HILDEGAZ trial.^57^ Following a haploidentical allogeneic stem cell transplant for hematological malignancies, 16 trial participants received ZOL and increasing doses of IL‐2. A significantly higher median number of Vγ9Vδ2 T‐cells was observed during IL‐2 treatment compared with non‐randomized transplant recipients who had not received ZOL/IL‐2. No Grade 3 adverse events (AEs) other than acute GVHD (which resolved after steroid treatment) were observed in the trial cohort. An alternative approach to stimulating γδ T cells in vivo involves the administration of a humanized BTN3A antibody (ICT01), which stimulates Vγ9Vδ2 T cells.^30^ The ongoing EVICTION Trial of ICT01 includes a cohort of patients with hematological malignancies, but the results of this group have yet to be reported. Interim results of ICT01 in combination with pembrolizumab in a cohort of patients with checkpoint inhibitor‐refractory melanoma suggested rapid trafficking of activated Vγ9Vδ2 T‐cells and transient, dose‐dependent increases in serum cytokines, and an overall favorable safety profile.^58^, ^59^ Furthermore, a novel single‐domain bispecific antibody (LAVA‐051),^60^ which engages tumor‐associated CD1d with the Vδ2 TCR was evaluated in a Phase I trial (NCT04887259). Preliminary results^61^, ^62^ of the trial involving multiple myeloma (MM) and CLL patients indicated safety and tolerability of LAVA‐051 at the evaluated doses; however, the trial has been terminated by the sponsors for commercial reasons.

### Adoptive γδ T‐cell therapy trials

The safety and feasibility of allogeneic γδ T cells expanded from healthy donors was investigated in Phase 1 TCB‐202‐001 (NCT03790072) Trial.^63^ Following a standard lymphodepleting conditioning regimen, trial participants with AML ineligible for allogeneic SCT received Vγ9Vδ2 γδ T cells, which had been expanded from haploidentical donors with ZOL and IL‐2. Three dose levels of γδ T cells, 10^6^/kg, 10^7^/kg, and 10^8^/kg, were evaluated with no GVHD or treatment‐related AEs of Grade 3 or greater. Although the trial was terminated early due to the COVID‐19 pandemic, response assessments suggested some preliminary evidence for efficacy in AML. One of the four patients who had a bone marrow assessment on Day 28 post‐cell infusion achieved a complete response (CR). Other trials involving the administration of allogeneic γδ T cells have confirmed the safety of this approach with no GVHD observed in a prior pilot study^64^ and in recent studies.^64^, ^65^

Chimeric antigen receptor (CAR)‐transduced and other modified γδ T cells are currently in early‐phase clinical trials. ADI‐001, an allogeneic Vδ1 CAR T‐cell therapy product targeting CD20, is being evaluated against B‐cell malignancies (NCT04735471, GLEAN‐1 study). Early results from the Trial which comprised 50% of patients who had progressed on previous CD19‐directed CAR T‐cell therapies, indicated ORR and CR rates of 71% and 63%, respectively,^66^ and robust in vivo expansion of ADI‐001 with levels significantly higher in trial subjects achieving a best overall response of PR or better.^67^ There was no association between the degree of patient–donor HLA mismatch and response, cell expansion, or persistence by Day 28. Given the safety profile and early signal of efficacy of ADI‐001, it is understood that the trial sponsors are considering a pivotal Phase 2 Trial. CAR‐modified γδ T‐cell products targeting CD19, CD7, and NKG2DL^68^ are also being investigated in early‐phase trials. An alternative T‐cell‐redirected strategy relevant to γδ T‐cell antitumor reactivity is the use of TCR transfer, an approach originally developed by Gadeta Therapeutics. This strategy is being investigated in a trial of γδTCR transduced T cells (NCT04688853) in patients with relapsed/refractory myeloma. In the Phase I study, trial participants receive autologous (mainly αβ) T cells transduced with a high‐affinity Vγ9Vδ2 TCR with broad antitumor reactivity.^69^

The role of adoptive transfer of γδ T cells to prevent AML recurrence post‐allogeneic SCT is being explored in several clinical trials with strategies including transfer of γδ T‐ and natural killer (NK)‐cell‐enriched (αβ T‐cell depleted) donor lymphocyte infusion (DLI; NCT05686538, IDEAL trial) and infusion of allograft donor‐derived ex vivo activated γδ T cells (NCT03533816 and NCT05015426). The administration of Expanded/Activated Gamma‐Delta (EAGD, also termed INB‐100) T‐cell infusions following haploidentical hemopoietic SCT for hematological malignancies is being investigated in a Phase I trial (NCT03533816) with early reports indicating favorable safety and tolerability profiles.^70^ Recent updated results released by IN8bio indicated remarkable clinical responses in the AML cohort with 100% of trial participants remaining in complete remission after a median follow‐up of 20.1 months with the observation of γδ T‐cell persistence beyond 12 months.^71^ In another Phase I trial of adoptive γδ T cells (NCT05015426), the published interim safety report indicated no GVHD, CRS, or immune effector cell‐associated neurotoxicity syndrome (ICANS) within the 42‐day dose‐limiting toxicity period at the two dose levels assessed (6.25 × 10^6^ cells/kg and 3.13 × 10^7^ cells/kg). The γδ T cells (>90% Vδ2) infused were obtained from the same stem cell donors, expanded ex vivo with ZOL and IL‐2, and further cultured with artificial antigen‐presenting cells. All trial participants were in CR (minimal residual disease negative) at the time of reporting, and updated results are awaited.

Currently, the majority of γδ T‐cell‐based adoptive therapy studies are early‐phase clinical trials, which are limited by small and often nonrepresentative patient populations. Although these trials provide proof of safety and tolerability for γδ T‐cell‐based approaches, large Phase III trials investigating useful efficacy endpoints such as long‐term persistence, response rates, and disease‐free survival are required to contextualize the therapeutic potential of γδ T cells relative to current conventional T‐cell‐based approaches.

## γδ T CELLS IN IMMUNOTHERAPY: CONTROVERSIES AND CHALLENGES

Studies have identified Vδ1 γδ T cells as the predominant subset in breast,^35^ colorectal,^72^ gastric,^73^ and melanoma^74^ tumor biopsies. Although most experimental models demonstrate potent antitumor properties and positive correlation with survival, one study identified a subset of γδ T cells (predominantly of the Vδ1 phenotype producing IL‐17, γδT17) which was associated with tumor progression in colorectal cancer by recruiting myeloid‐derived suppressor cells.^72^ Although the mechanism of polarization of γδ T cells towards the IL‐17‐producing phenotype within human tissues remains unclear, a distinct subset of murine γδ T cells that expressed the IL‐23 receptor responded to IL‐1β and IL‐23 stimulation leading to the production of IL‐17, independent of TCR stimulation.^75^ The IL‐23‐driven γδT17 differentiation of peripheral γδ T cells was also confirmed in another murine model.^76^ Furthermore, upon activation, Vδ1 T cells from adjacent healthy colorectal tissues produced IFNγ but hardly any IL‐17 or TNFα in contrast to Vδ1 cells within colorectal cancer biopsies.^77^ In another study, increased intratumoral frequencies of IL‐17‐producing Vδ1 and Vδ2 T cells were associated with advanced skin squamous cell carcinoma (SCC) and risk of mortality in contrast to IFNγ‐producing intratumoral Vδ1 and Vδ2 T cells, which were more frequent in SCC patients at earlier disease stages.^78^ Recently, using a combination of RNA sequencing and flow cytometry methods, a subset of γδ T cells (mainly Vδ1 and Vδ3) producing amphiregulin (AREG) was identified in colorectal cancer samples in association with a “wound healing,” pro‐tumor phenotype.^79^ Notably, the study also found (in contrast to previous studies) that neither Vδ1 nor Vδ2 cells produced IL‐17; rather Vδ3 T cells were the source of IL‐17 in their model. Another recently published study also found no *IL17A*‐expressing Vδ1 T cells on transcriptomic analyses of skin‐resident γδ T cells.^36^ From these experimental observations, γδT17 induction and the pro‐inflammatory γδ T‐cell phenotype appear to be context and tissue‐dependent, likely driven by conditions within the TME. These findings highlight the need for detailed phenotypic characterization of γδ T‐cell products for clinical development.

The main obstacle to the development of γδ T‐cell immunotherapy has been the difficulty in expanding this relatively rare T‐cell subset to clinically useful cell numbers. The earliest methods employed T‐cell mitogens such as PHA and Con A, which made clinical translation difficult. The Vγ9Vδ2 subset is usually expanded from PBMCs with ZOL and stimulatory cytokines, but there are often significant variations in expansion efficiency between donors. While IL‐2 and IL‐15 have typically been used in expanding γδ T cells, novel combinations of cytokines have been investigated. A recent study demonstrated increased Vγ9Vδ2 T‐cell yield and enhanced effector functions against AML when γδ T cells were expanded in culture media containing transforming growth factor‐β (TGF‐β).^80^ Alternative methods include the use of agonist γδTCR antibodies; however, an argument against this method is that chronic stimulation via the TCR during the expansion process may lead to functional exhaustion. Both artificial APCs and feeder cell‐free methods have been evaluated in the expansion of γδ T cells^81^ and new clinically relevant, Good Manufacturing Practice (GMP)‐grade methods being developed for the expansion of the rarer Vδ1 subset from PBMCs include the depletion of αβ T cells followed by expansion with anti‐CD3 (OKT3) and IL‐15.^50^ The Delta one T (DOT) cell protocol which uses a combination of cytokines (IL‐4, IFNγ, IL‐21, and IL‐1β) and antiCD3 mAb (OKT3) expanded Vδ1 T cells which were cytotoxic against cancer cell lines.^49^ An allogeneic cell therapy product (GDX012), based on the DOT protocol, is currently in clinical trials in patients with AML.^82^ A 2023 paper described a protocol using IL‐15, IL‐18, anti‐CD3, and anti‐CD2 promoted the expansion of Vδ1 T cell with a strong antitumor phenotype.^79^ With the recent interests in γδ T‐cell‐based therapeutics, it is crucial to advance research into the fundamental biology of these cells and the multifaceted functions of γδ T‐cell subsets particularly their interaction with the tumor microenvironment. Further research priorities should be directed at understanding the basis for the significant variation in expansion efficiency between donors with a view to determining predictive factors for improved γδ T‐cell expansion and quality.

T‐cell exhaustion in conventional αβ T cells is well characterized, but emerging evidence suggests that canonical exhaustion markers such as PD‐1 do not define exhaustion in γδ T cells as functional Vδ1 cells often express high levels of PD‐1. Therefore, basic research into the characterization of γδ T‐cell exhaustion signatures at the transcriptomic level is crucial to the identification of γδ T‐cell‐specific exhaustion markers, which will ultimately lead to the development of optimal cell products for the clinic.

## CONCLUSIONS

### γδ T‐cell therapy in hematology: Current gaps and potentials

Current CAR T‐cell strategies involve manipulation of autologous T cells, and the manufacturing process often takes several weeks. This is unsuccessful in up to 25% of patients due to poor T‐cell fitness, effect of prior therapies, and suboptimal constitution of PBMCs.^83^ In patients with rapidly progressing disease and those with T‐cell dysfunction as a result of prior lymphotoxic treatments, autologous cell therapy strategies are undesirable; hence, the need for the development of allogeneic healthy donor‐derived cell therapy products. The use of unconventional T cells (γδ T cells, iNKT, and mucosal‐associated invariant T [MAIT] cells)^84^, ^85^ as well as innate immune cells such as NK cells as CAR effectors provides an attractive strategy given their broad anticancer reactivity and lack of alloreactivity. These immune cells offer the potential for “off‐the‐shelf” cell therapy products, which not only minimize the time to treatment but also potentially guarantee a standardized cell product. A summary of some immune effector cells being developed for adoptive cell therapy is shown in Table 3.

**Table 3:** Comparison of immune effector cells for adoptive cell therapy.

	γδ T cells	αβ T cells	NK cells	iNKT cells
Anticancer activity of unmanipulated cells	Yes; MHC‐presentation not required	Yes; requires antigen presentation in the context of self‐MHC molecules	Yes; recognizes stress signals via innate NKRs	Yes; CD1d‐restricted. Classical MHC presentation not required
Frequency in PB	Uncommon	Abundant	Less frequent	Rare
Ease of manufacture	+	+++	+	+
Feeder/antigen‐presenting cell requirement for expansion	Not essential	May require	Often requires	Often requires
Alloreactivity	No	Yes	No	No
Cell manipulation strategies in clinical trials	CAR, TCR transfer (TCRt)	CAR T	CAR‐NK	CAR‐iNKT
“Off‐the‐shelf” potential	Yes	No; unless TCR deleted	Yes	Yes
In vivo persistence	Prolonged^a^	Variable	Limited	Prolonged^a^
Risk of cytokine release syndrome (CRS)	Low	Moderate/high	Low	Low
Hematological conditions for CAR‐based therapies (clinical trials or licensed)	B‐cell malignancies Multiple myeloma T‐cell malignancies AML	B‐cell lymphoma/leukemia Multiple myeloma	Multiple myeloma B‐cell malignancies	B‐cell lymphoma/leukemia
Currently approved cell therapy products^b^	—	7	—	—
Limitations	Limited knowledge of most γδ TCR ligands. Variable donor expansions. Possible regulatory function.	Mostly limited to autologous use. Potential for T cell exhaustion. Limited activity in solid tumors.	Limited persistence in in vivo models. Lacks TCR‐based clonal expansion.	Rare immune cells. Potentially difficult to expand.

Abbreviations: γδ, gamma delta; AML, acute myeloid leukemia; CAR, chimeric antigen receptor; MHC, Major Histocompatibility Complex; NK, natural killer; NKR, natural killer receptor; PB, peripheral blood; TCR, T‐cell receptor.

a Based on limited clinical data.

^b^ As of May 2025.

The feasibility of the off‐the‐shelf approach for γδ T cells is evidenced by the fact that clinical trials involving allogeneic γδ T cells have not reported GVHD as a complication.^63^, ^86^ Specifically, γδ T cells possess characteristics that are ideal for adoptive cell therapy. Besides their lack of alloreactivity, γδ T‐cell‐based CAR therapy has been associated with a lower risk of CRS compared with conventional αβ T cells,^87^ and data from a Phase I trial indicated prolonged persistence in vivo.^88^ Furthermore, given the natural localization of certain γδ T‐cell subsets to tissues, it is thought that γδ T cells would be effective in the solid tumor microenvironment, hence the recent increase in γδ T‐cell trials against solid tumors. The tissue tropism of γδ T cells would also be crucial in certain hematological malignancies, for instance, in extramedullary myeloma, where optimal immune cell infiltration is required for tumor eradication. Key limitations in the use of γδ T cells for adoptive cell therapy include donor variability in γδ T‐cell subsets and expansion, limited knowledge of the natural ligands of the γδ TCR, and their diverse functions in disease pathogenesis.

In the post‐allogeneic SCT setting, the administration of DLIs is currently an established therapy for mixed donor chimerism^89^ but this is often associated with an increased risk of GVHD. The lack of MHC‐restriction by γδ T cells potentially provides an opportunity to achieve GvL effects without the risk of GVHD in the post‐allogeneic SCT setting. Therefore, the use of ex vivo expanded donor‐derived γδ T cells post‐allogeneic SCT would be a rational strategy to deepen clinical responses and as an effective therapy against post‐transplant infections.

The hematology cell therapy space has seen tremendous progress with all seven autologous FDA‐approved CAR T‐cell products, being for B‐cell malignancies and MM.^90^ Despite these advancements, there are still significant unmet needs for advanced cell therapies against many hematological malignancies. For instance, there are currently limited treatment options for relapsed/refractory AML, and the lack of an ideal AML‐associated antigen limits the development of safe CAR‐based therapy. Given the ample preclinical evidence of unmanipulated γδ T‐cell activity against AML,^91^ the use of ex vivo allogeneic γδ T cells could be an effective therapeutic strategy for AML as well as other malignancies where no ideal tumor target exists. The anticancer activity of unmanipulated γδ T cells in this setting is underpinned by the recognition of neoantigens and “stress signals” by the myriad of innate receptors and the γδTCR. However, tumor specificity and anticancer efficacy are enhanced by CAR‐based approaches, and this is preferable where an ideal target antigen exists. To improve efficacy and ensure γδ T‐cell persistence in vivo, next‐generation γδ CAR engineering approaches will require a combination of strategies including multi‐antigen targeting, novel CAR‐binding domains, and cytokine armoring. Given the pivotal role of IL‐15 and other stimulatory cytokines in the expansion and maintenance of γδ T cells, the development of cytokine‐armoured CARs is expected to improve persistence in vivo. However, concerns around cytokine‐induced uncontrolled T‐cell proliferation and other toxicities need to be addressed with novel strategies including inducible cytokine expression or the incorporation of a suicide gene in the CAR construct.

### Future perspectives

The recent explosion of scientific interest in γδ T cells reflects the realization of their crucial role as a first line of defense against certain microbes and transformed cells. Despite the increased γδ T‐cell research focus, many fundamental questions remain unanswered. These include an understanding of the factors underlying specific tissue localization of γδ T‐cell subsets, identification of γδ TCR ligands, and the complex roles of γδ T cells in health and disease pathogenesis. The considerable experimental evidence for γδ T‐cell antitumor activity against a broad range of solid and hematological cancers makes γδ T cells ideal candidates for translation into cell therapy products for use in the clinic. However, there is a need for optimization of γδ T‐cell expansion methods with a view to ensuring adequate T‐cell fitness and avoidance of undesirable immune cell populations. As knowledge of the biology of γδ T‐cell subsets advances, it is likely that new expansion methods will be developed that optimize desirable effector functions.

While unmanipulated γδ T cells demonstrate potent effector functions against tumors in experimental models, further enhancement and redirection of function are achieved by transduction of CARs.^92^, ^93^ Feasibility for this approach in the clinic has been demonstrated in prostate, triple negative breast, and hepatocellular preclinical models.^94^, ^95^ So far, early results from a clinical trial of allogeneic CD20 CAR γδ T cells have demonstrated safety and tolerability in a heavily pretreated group of patients with B‐cell malignancies.^86^ Another cell manipulation strategy currently investigated in a clinical trial is γδTCR transfer (NCT04688853), which involves transduction of a specific γδ TCR onto autologous αβ T cells. In addition to current established CAR redirection strategies, research efforts are being channeled into strategies that optimize persistence and effector functions. These include the arming of CAR T cells with cytokines^80^, ^96^, ^97^ and the conditional activation of cytokine signaling following CAR engagement and granzyme release.^98^ It is expected that the γδ T‐cell therapy field will see an expansion of advanced cell manipulation strategies in the future. Cell‐based immunotherapy against solid tumors is currently limited by several unique obstacles including impaired immune cell infiltration due to the immunosuppressive tumor microenvironment. As γδ T‐cell subsets have tissue distributions likely indicating adaptations to specific tissues, it is expected that γδ T cells would be ideal immune effectors against solid tumors either alone or in combination with checkpoint inhibitors. Evidently, well‐designed clinical trials are required to determine the efficacy of γδ T cells in solid cancers, and the results of ongoing trials are awaited.

In summary, γδ T cells are versatile immune cells with immense potential for development as cell therapy products against a broad range of cancers; however, further fundamental research is required to fully elucidate the complex roles of γδ T cells in cancer. A combination of detailed phenotypic characterization of γδ T cells, optimized expansion methods, and cell manipulation strategies would likely lead to the development of highly effective γδ T‐cell therapy products. While most recent advancements in cell therapy, particularly CAR T‐cell therapy, have been hematology‐led, it is anticipated that γδ T cells would also open new avenues for the development of adoptive cell therapies for solid tumors in the near future.

## AUTHOR CONTRIBUTIONS

**Charles Agbuduwe**: Conceptualization; methodology; writing—original draft; writing—review and editing; data curation; resources. **John Maher**: Writing—review and editing; supervision; methodology. **John Anderson**: Writing—review and editing; supervision; methodology.

## CONFLICT OF INTEREST STATEMENT

C.A.: no conflicts. J.M.: chief scientific officer, scientific founder, and shareholder of Leucid Bio. J.A.: founder stock in Autolus Ltd. and holds patents related to CAR‐T technology.

## CONSENT FOR PUBLICATION

Not applicable.

## ETHICS STATEMENT

Not applicable.

## FUNDING

C.A. is funded by an NIHR Imperial Biomedical Research Centre award (PB1240) as part of a research fellowship. J.A. is supported in part by the NIHR Great Ormond Street Biomedical Centre. No funders were involved in the conceptualization, design, decision to publish, or preparation of the manuscript.

## Data Availability

Data sharing is not applicable to this article as no datasets were generated or analyzed during the current study.
